# Evidence for progressive reduction and loss of telocytes in the dermal cellular network of systemic sclerosis

**DOI:** 10.1111/jcmm.12028

**Published:** 2013-02-27

**Authors:** Mirko Manetti, Serena Guiducci, Martina Ruffo, Irene Rosa, Maria Simonetta Faussone-Pellegrini, Marco Matucci-Cerinic, Lidia Ibba-Manneschi

**Affiliations:** a Department of Anatomy, Histology and Forensic Medicine, University of FlorenceFlorence, Italy; b Department of Biomedicine, Division of Rheumatology, Azienda Ospedaliero-Universitaria Careggi, Excellence Centre for Research, Transfer and High Education DENOthe, University of FlorenceFlorence, Italy

**Keywords:** telocytes, skin, dermis, systemic sclerosis, scleroderma, fibrosis, immunohistochemistry, transmission electron microscopy

## Abstract

Telocytes, a peculiar type of stromal cells, have been recently identified in a variety of tissues and organs, including human skin. Systemic sclerosis (SSc, scleroderma) is a complex connective tissue disease characterized by fibrosis of the skin and internal organs. We presently investigated telocyte distribution and features in the skin of SSc patients compared with normal skin. By an integrated immunohistochemical and transmission electron microscopy approach, we confirmed that telocytes were present in human dermis, where they were mainly recognizable by their typical ultrastructural features and were immunophenotypically characterized by CD34 expression. Our findings also showed that dermal telocytes were immunophenotypically negative for CD31/PECAM-1 (endothelial cells), α-SMA (myofibroblasts, pericytes, vascular smooth muscle cells), CD11c (dendritic cells, macrophages), CD90/Thy-1 (fibroblasts) and c-kit/CD117 (mast cells). In normal skin, telocytes were organized to form three-dimensional networks distributed among collagen bundles and elastic fibres, and surrounded microvessels, nerves and skin adnexa (hair follicles, sebaceous and sweat glands). Telocytes displayed severe ultrastructural damages (swollen mitochondria, cytoplasmic vacuolization, lipofuscinic bodies) suggestive of ischaemia-induced cell degeneration and were progressively lost from the clinically affected skin of SSc patients. Telocyte damage and loss evolved differently according to SSc subsets and stages, being more rapid and severe in diffuse SSc. Briefly, in human skin telocytes are a distinct stromal cell population. In SSc skin, the progressive loss of telocytes might (i) contribute to the altered three-dimensional organization of the extracellular matrix, (ii) reduce the control of fibroblast, myofibroblast and mast cell activity, and (iii) impair skin regeneration and/or repair.

## Introduction

Systemic sclerosis (SSc), or scleroderma, is a chronic connective tissue disease of unknown origin characterized by widespread microvascular damage, immune dysregulation with production of autoantibodies and progressive fibrosis affecting the skin and multiple internal organs, including the lung, heart, kidney and gastrointestinal tract 1–4. Endothelial cell injury is supposed to be the initial event which together with inflammatory and autoimmune reactions leads to the chronic activation and transdifferentiation of fibroblasts into myofibroblasts, finally resulting in a deregulated wound healing process and severe tissue fibrosis 1, 2, 5. The overproduction and accumulation of collagen and other extracellular matrix components ultimately disrupt the architecture of the affected tissues and frequently lead to organ dysfunction and failure 1, 2. Moreover, the concomitant fibroproliferative vasculopathy, characterized by subendothelial intimal fibrosis affecting small and medium-sized arteries and arterioles, and the loss of microvessels may lead to chronic tissue ischaemia with severe vascular complications, such as digital ulceration and gangrene 6, 7.

In early SSc, the main cutaneous histopathological features are represented by perivascular inflammatory infiltrates composed of monocytes and lymphocytes, by dermal oedema and by a variable extent of extracellular matrix accumulation in the papillary and reticular dermis 1, 8, 9. In advanced SSc, severe dermal fibrotic changes with tightly packed and irregularly distributed collagen bundles, flattening of dermal papillae, loss of capillaries, occlusion of arterioles, damage of nerve fibres, and atrophy of skin appendages are commonly observed 1, 8–10.

Up to now, most of the studies have focused on the role of the fibroblasts in the pathogenesis of SSc. It is well known that fibroblasts are dysregulated and activated by a number of profibrotic cytokines, growth factors and stimulatory autoantibodies, and that they transform into myofibroblasts and produce an excessive amount of types I, III, VI and VII collagen, fibronectin, elastin and proteoglycans 1, 2, 11, 12. Abnormal SSc fibroblasts are believed to develop from a subset of cells that have escaped from normal control mechanisms. Indeed, fibroblasts from clinically affected SSc skin cultured *in vitro* still continue to produce excessive amounts of extracellular matrix proteins, suggesting that once activated, these cells establish a constitutive self-activation system 12, 13. However, little is known about the possible involvement of other stromal cell types in SSc pathophysiology.

Telocytes, formerly called interstitial Cajal-like cells, are a distinct population of stromal (interstitial) cells which have been recently identified in a wide variety of human and mammalian tissues and organs (http://www.telocytes.com) 14, 15. Popescu and Faussone-Pellegrini have thoroughly described the peculiar ultrastructural phenotype and the immunophenotype of the telocytes 14, 16. Telocytes are ultrastructurally characterized by a small cell body and extremely long processes, termed telopodes, which display a moniliform aspect alternating thin segments (podomers) with dilated regions (podoms) 16. Moreover, telocytes have been described to possess different immunophenotype markers, such as the sialylated transmembrane glycoprotein CD34 and the tyrosine kinase receptor c-kit/CD117, among a variety of cavitary and non-cavitary organs, and even within the same organ examined 16. Currently, the presence of telocytes has been identified in the heart 17–19, gut 20, 21, placenta 22, urinary tract 23, lungs 24, 25, pleura 26, exocrine pancreas 27, salivary glands 28, mammary glands 29, myometrium 30 and skeletal muscle 31. Telocytes have also been recently described in human dermis, where they may participate to skin homeostasis and remodelling 32, 33.

The aim of the present work was to investigate the presence, the distribution, the immunophenotype and the ultrastructural features of telocytes in normal skin and in clinically involved skin from different subsets and stages of SSc.

## Materials and methods

### Patients, controls and skin samples

Full-thickness skin biopsies were obtained from the clinically involved skin of one-third of the distal forearm of 24 patients with SSc (20 women, 4 men; mean ± SD age: 46 ± 13 years) recruited from the Division of Rheumatology, University of Florence. Patients were classified as having limited cutaneous SSc (lcSSc; *n* = 13) or diffuse cutaneous SSc (dcSSc; *n* = 11) according to LeRoy *et al*. 34. Disease duration was calculated from the time of onset of the first clinical manifestation of SSc (other than Raynaud's phenomenon). Patients were further classified as being in the early stage (*n* = 11, 5 lcSSc, 6 dcSSc) or advanced stage (*n* = 13, 8 lcSSc, 5 dcSSc) of SSc according to disease duration (early lcSSc, disease duration <5 years; early dcSSc, disease duration <3 years) and skin histopathology as previously described 9. We considered clinically involved skin for values of skin thickness ≥2, according to the modified Rodnan skin thickness score 8, 35. In SSc, the skin score evaluates the thickness of skin as assessed by clinical palpation of 17 body areas on a scale of 0–3 (0 = normal, 1 = mild thickening, 2 = moderate thickening, 3 = severe thickening), and from the sum of the scores from all body areas, with a maximum possible total score of 51 35. In a subgroup of lcSSc patients (*n* = 4), biopsies were also taken from clinically non-involved skin. None of the patients was receiving immunosuppressive medication or other potentially disease-modifying drugs at the time of skin biopsy. All patients with SSc underwent a 15-day treatment washout before skin biopsy was performed. During this period, only proton-pump inhibitors were allowed. Patients who could not undergo washout due to severe organ complications were not enrolled in the study. Skin samples from the same forearm region of 10 age- and sex-matched healthy donors were used as controls (8 women, 2 men; mean ± SD age: 44 ± 16 years). All the volunteers signed an informed consent form, and the study complied with the principles of the Declaration of Helsinki and was approved by the local Institutional Review Board.

Each skin biopsy was divided into two specimens and processed for light microscopy and transmission electron microscopy respectively.

### Immunohistochemistry

#### Light microscopy

Skin biopsies were fixed in 10% buffered formalin, dehydrated in a graded ethanol series and xylene, and embedded in paraffin. Skin sections were cut (5 μm thick) using a Leica RM2255 rotary microtome (Leica Microsystems, Mannheim, Germany), deparaffinized and either stained with haematoxylin and eosin for routine histology or processed for double immunoenzymatic labelling using horseradish peroxidase (HRP) and alkaline phosphatase (AP) detection systems. For heat-induced antigen retrieval, skin sections were boiled for 10 min. in sodium citrate buffer (10 mM, pH 6.0) followed by cooling of the slides for 20 min. at room temperature in the same buffer. The sections were then washed three times in phosphate buffered saline (PBS) and subjected to double immunoenzymatic staining using the Cell & Tissue Staining Kit-HRP System for the detection of mouse primary IgG antibodies (catalogue no. CTS002, R&D Systems, Minneapolis, MN, USA) and the UltraVision AP Detection System for the detection of rabbit primary IgG antibodies (catalogue no. TR-015-AF, LabVision, Fremont, CA, USA) according to the manufacturer's protocol. Briefly, after treatment with Peroxidase Blocking Reagent (R&D Systems) for 5 min., the sections were washed in PBS and incubated with Serum Blocking Reagent G (R&D Systems) for 15 min., followed by a 15-min. incubation with Avidin Blocking Reagent (R&D Systems). The samples were then rinsed in PBS, treated with Biotin Blocking Reagent (R&D Systems) for 15 min. and subsequently incubated with a mixture of mouse monoclonal anti-human CD34 (1:50 dilution; clone QBEnd-10, catalogue no. M7165, Dako, Glostrup, Denmark) and rabbit polyclonal anti-human c-kit/CD117 (1:300 dilution; catalogue no. A4502, Dako) in a humidified chamber overnight at 4°C. Tissue sections were incubated sequentially with biotinylated goat antimouse secondary antibodies for 45 min. and High Sensitivity Streptavidin-HRP Conjugate (R&D Systems) for 30 min. at room temperature. CD34 immunoreactivity was developed using the Vina Green™ Chromogen Kit (catalogue no. VG807, Biocare Medical, Concord, CA, USA) according to the manufacturer's instructions. After the first colour reaction, the sections were washed three times with PBS and incubated with biotinylated goat anti-rabbit secondary antibodies for 10 min. followed by a 10-min. incubation with Streptavidin-AP complex (LabVision) at room temperature. The c-kit fuchsin-red reaction product was developed by using the Warp Red™ Chromogen Kit (catalogue no. WR806, Biocare Medical). Parallel sections were incubated with isotype- and concentration-matched normal IgG (Sigma-Aldrich, St. Louis, MO, USA) to replace the primary antibodies as negative staining controls. The immunolabelled sections were observed under a Leica DM4000 B microscope equipped with fully automated transmitted light and fluorescence axes (Leica Microsystems). Transmitted light images were captured using a Leica DFC310 FX 1.4-megapixel digital colour camera equipped with the Leica software application suite LAS V3.8 (Leica Microsystems).

#### Fluorescence microscopy

For antigen retrieval, paraffin-embedded skin sections (5 μm thick) were deparaffinized and boiled for 10 min. in sodium citrate buffer (10 mM, pH 6.0). The sections were washed three times in PBS, incubated in 2 mg/ml glycine for 10 min. to quench autofluorescence caused by free aldehydes, and then blocked for 1 hr at room temperature with 1% bovine serum albumin (BSA) in PBS. Double immunofluorescent stainings with mouse and rabbit antibodies were performed by mixing primary antibodies and subsequently mixing fluorochrome-conjugated secondary antibodies. The slides were incubated overnight at 4°C with the following primary anti-human antibodies diluted in PBS with 1% BSA: mouse monoclonal anti-CD34 (1:50 dilution; Dako), rabbit polyclonal anti-c-kit/CD117 (1:300 dilution; Dako), rabbit polyclonal anti-α-smooth muscle actin (α-SMA; 1:100 dilution; catalogue no. ab5694, Abcam, Cambridge, UK), mouse monoclonal anti-mast cell tryptase (1:50 dilution; catalogue no. ab2378, Abcam), rabbit monoclonal anti-CD11c (1:50 dilution; catalogue no. ab52632, Abcam), rabbit monoclonal anti-CD90/Thy-1 (1:50 dilution; catalogue no. ab92574, Abcam), and rabbit polyclonal anti-CD31/platelet-endothelial cell adhesion molecule*-*1 (PECAM-1; 1:50 dilution; catalogue no. ab28364, Abcam). After extensive washing in PBS, slides were incubated with secondary antibodies for 45 min. at room temperature in the dark. The immunoreactions were revealed using Alexa Fluor-488-conjugated goat antimouse IgG or Rhodamine Red-X-conjugated goat anti-rabbit IgG (1:200 dilution; Molecular Probes, Eugene, OR, USA) as secondary antibodies. Irrelevant isotype- and concentration-matched IgG (Sigma-Aldrich) were used as negative controls. Cross-reactivity of secondary antibodies was tested in control experiments in which primary antibodies were omitted. In some sections, the nuclei were counterstained with 1 μg/ml 4′,6-diamidino-2-phenylindole (DAPI; Sigma-Aldrich). Tissue sections were then mounted with an antifade aqueous mounting medium (Biomeda Gel mount, Electron Microscopy Sciences, Foster City, CA, USA) and examined with the Leica DM4000 B microscope (Leica Microsystems).

### Quantitative analysis

Quantitative analysis of telocytes and microvessels was performed on skin sections double-immunolabelled with the mouse monoclonal anti-CD34 and the rabbit polyclonal anti-CD31/PECAM-1 antibody and counterstained with DAPI for nuclei. CD34-positive/CD31-negative spindle-shaped cells (telocytes) and CD34/CD31-double-positive microvessels were counted in 10 randomly chosen microscopic high-power fields (hpf; 40× original magnification) of the papillary dermis and 10 hpf of the reticular dermis (excluding skin adnexal structures) per sample. Only the cells with well-defined nuclei were counted. The same procedure was used for the quantification of fibroblasts on skin sections immunolabelled with the rabbit monoclonal anti-CD90/Thy-1 antibody and counterstained with DAPI for nuclei. Counting was performed by two independent observers who were blinded with regard to the skin biopsy classification. The final result was the mean of the two different observations for each sample. Data are represented as mean ± SD and were analysed using the Student's *t-*test for independent samples assuming equal variances. *P* < 0.05 was considered statistically significant.

### Transmission electron microscopy

For transmission electron microscopy, skin specimens were divided into small fragments and fixed in 4% cacodylate-buffered glutaraldehyde (pH 7.4) at room temperature. Then, the specimens were rinsed in a cacodylate-buffered solution supplemented with sucrose, post-fixed in 1% osmium tetroxide (Electron Microscopy Sciences), dehydrated with graded alcohol series, clarified in propylene and embedded in epoxy resin (Epon 812). Semithin sections (2 μm thick) were cut with a RMC MT-X ultramicrotome (EMME3, Milan, Italy) and stained with a solution of toluidine blue in 0.1 M borate buffer, and then observed under a light microscope. Ultrathin sections (∼70 nm thick) of the selected areas were obtained with the same ultramicrotome using a diamond knife and stained with an alcoholic solution of uranyl acetate, followed by an alkaline solution of bismuth subnitrate. All the ultrathin sections were examined under a JEOL 1010 electron microscope (Jeol, Tokyo, Japan) at 80 kV and photographed.

## Results

### Light and fluorescence microscopy

As CD34 and c-kit antigens have been described as immunohistochemical markers of telocytes in humans and rodents 16, a double immunostaining for CD34 and c-kit was performed to investigate their expression patterns in the skin of healthy individuals and SSc patients.

#### Normal skin

**Fig. 1:**
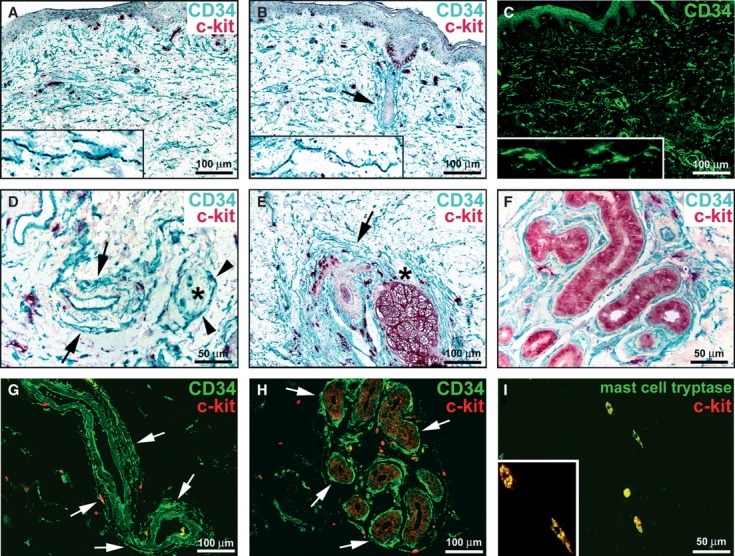
Normal skin, immunohistochemistry. (**A**, **B**, **D**–**F**) Double immunoenzymatic labelling for CD34 and c-kit with horseradish peroxidase (green) and alkaline phosphatase (red) detection systems respectively. (**C**) CD34 immunofluorescence labelling. (**G** and **H**) Double immunofluorescence labelling for CD34 (green) and c-kit (red). (**I**) Double immunofluorescence labelling for mast cell tryptase (green) and c-kit (red). (A–H) Dermal interstitial cells, presumably telocytes and endothelial cells display CD34-immunoreactivity. The CD34-positive cells do not display c-kit-immunoreactivity. In the papillary and reticular dermis, telocytes form an interstitial network (A–C). Telocytes have an elongated shape with an oval or triangular body and two or three long and thin cell processes with knobs/dilations along their length (A–C, higher magnification view in *insets*). Telocytes form a thin and almost continuous layer encircling arterioles (D and G, *arrows*). A nerve [denoted by an *asterisk* in (D)] is surrounded by telocytes (*arrowheads*). Telocytes make an almost continuous multilayered sheath around hair follicles (B and E, *arrows*), sebaceous glands (E, *asterisk*) and the secretory and excretory portions of eccrine sweat glands (F and H, *arrows*). Many c-kit-positive cells, round or oval in shape, are observed in the dermis, often in close contact with telocytes (A, B, D-H). Melanocytes (B) and epithelial cells of sebaceous (E) and sweat (F, H) glands are c-kit-immunoreactive. (I) The c-kit-positive cells show mast cell tryptase immunoreactivity. A higher magnification view of two double-positive mast cells is shown in the *inset*. Scale bars are indicated in each panel.

In normal skin biopsies, both immunoenzymatic and immunofluorescence labelling revealed numerous CD34-positive cells throughout the whole dermis (Fig. 1A–H). Some of these CD34-positive cells were endothelial cells of capillary vessels and arterioles, and many others were stromal (interstitial) cells (Fig. 1A–C). Consistent with recent findings from Popescu group 33, these stromal cells were presumably telocytes as they appeared as spindle-shaped cells with a small body and very long and thin prolongations forming a labyrinthine interstitial network in the papillary and reticular dermis (Fig. 1A–C). These cells (hereafter referred to as telocytes) appeared polymorphic, but had in common the elongated shape with an oval or triangular body and a variable number of cell processes, frequently two or three (Fig. 1A–C, insets). Small knobs/dilations, oval or triangular in shape, were present along the processes (Fig. 1A–C, insets). The majority of telocytes were distributed among dermal collagen bundles, retinacula cutis and adipocytes in the hypodermis, some surrounded capillary vessels and others formed a thin and almost continuous layer encircling arterioles and nerves (Fig. 1A–D and G). Moreover, some of these cells were concentrated around skin adnexal structures, making an almost continuous multilayered sheath around hair follicles, arrector pili muscle bundles, sebaceous glands and both the secretory and excretory portions of eccrine sweat glands (Fig. 1B, E, F and H).

Everywhere in the dermis, the CD34-positive cells identified as telocytes did not display c-kit-immunoreactivity (Fig. 1A, B and D–H). However, there were many c-kit-positive cells which were identifiable as mast cells, as they were round or oval in shape (Fig. 1A, B and D–H). Moreover, these cells exhibited tryptase immunoreactivity, thus confirming that they were mast cells (Fig. 1I). Mast cells were often observed in close contact with telocytes (Fig. 1A, B and D–H). In addition, melanocytes in the basal layer of the epidermis and epithelial cells of sebaceous and sweat glands were c-kit-immunoreactive (Fig. 1B, E, F and H).

**Fig. 2:**
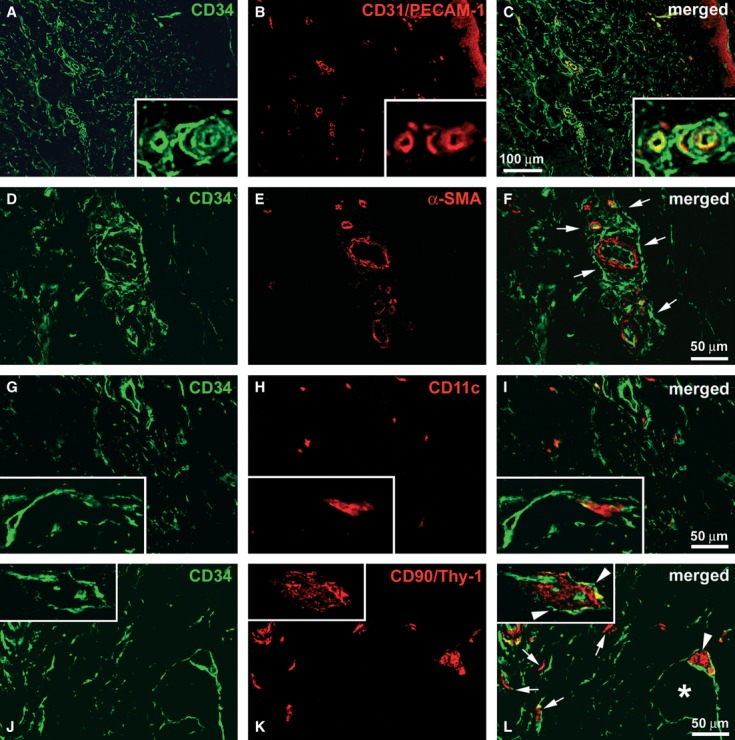
Normal skin, immunohistochemistry. Double immunofluorescence labelling for CD34 (green) and (**A**–**C**) CD31/platelet-endothelial cell adhesion molecule*-*1 (PECAM-1, red), (**D**–**F**) α-smooth muscle actin (α-SMA, red), (**G**–**I**) CD11c (red), (**J**–**L**) CD90/Thy-1 (red). (A–C) Endothelial cells are CD34/CD31-double-positive, while telocytes are CD34-positive/CD31-negative. The *inset* is a higher magnification view showing microvessels surrounded by telocytes. (D–F) Telocytes do not display α-SMA-immunoreactivity. α-SMA-positive pericytes and vascular smooth muscle cells are surrounded by telocytes (F, *arrows*). (G–I) Telocytes are CD11c-negative, while dendritic cells and macrophages show CD11c-immunoreactivity. A dendritic cell is embraced by the long processes of a telocyte (I, *inset*). (J–L) Telocytes do not display immunoreactivity for CD90/Thy-1, while fibroblasts are CD90-positive/CD34-negative (L, *arrows*). Telocyte prolongations surround a CD90-positive stem cell niche (L, *arrowhead*) near a sebaceous gland (L, *asterisk*) and a vascular wall-resident stem cell niche (L, *inset*, *arrowheads*). Scale bars are indicated in each panel.

Double immunofluorescence labelling for CD34 and the pan-endothelial cell marker CD31/PECAM-1 further allowed to ascertain that the CD34/CD31-double-positive cells were endothelial cells, while the CD34-positive/CD31-negative cells were telocytes, the latter representing the majority of CD34-expressing dermal cells (Fig. 2A–C). Double labelling also showed that telocytes did not display immunoreactivity for either α-SMA, unlike myofibroblasts, pericytes of capillary vessels and vascular smooth muscle cells of arterioles (Fig. 2D–F), or CD11c, a molecular marker of dendritic cells and macrophages (Fig. 2G–I). Interestingly, the CD11c-positive dendritic cells were often embraced by the long and thin processes of telocytes (Fig. 2I, inset). Finally, telocytes could be clearly distinguished from fibroblasts, because they did not express the CD90/Thy-1 marker which, as expected, was highly expressed in fibroblasts (Fig. 2J–L). Telocytes and fibroblasts were often spotted in the vicinity to each other (Fig. 2L). Moreover, telocyte prolongations surrounded the niches of CD90-positive stem cells located close to the hair follicles and sebaceous glands, as well as the so-called perivascular or vascular wall-resident stem cell niches 36–39 (Fig. 2L, inset).

#### SSc skin

**Fig. 3:**
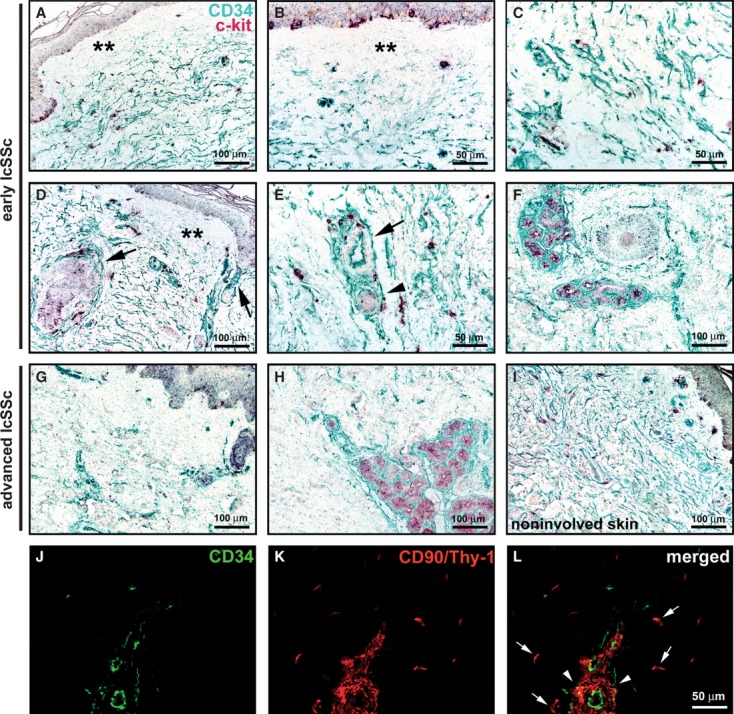
Limited cutaneous systemic sclerosis (lcSSc) skin, immunohistochemistry. (A–I) Double immunoenzymatic labelling for CD34 and c-kit with horseradish peroxidase (green) and alkaline phosphatase (red) detection systems respectively. Everywhere in the dermis, telocytes and endothelial cells are CD34-positive, mast cells are c-kit-positive. Melanocytes (A, B, D, G, I) and epithelial cells of sebaceous (D and G) and sweat (F and H) glands are c-kit-immunoreactive. (A–F) Early lcSSc skin. Telocytes are absent from the papillary dermis (A, B, D, *double asterisks*) and patchily reduced in the reticular dermis. In the reticular dermis, most telocytes are enlarged in shape (C). Telocytes are present around arterioles (E, *arrow*), nerves (E, *arrowhead*), hair follicles and sebaceous glands (D, *arrows*), and eccrine sweat glands (F). (G–I) Advanced lcSSc skin. Telocytes are absent from the papillary dermis and severely reduced in the reticular dermis and around most of the adnexal structures (G). In the deep reticular dermis, telocytes are preserved around sweat glands (H). A normal distribution of telocytes is observed in clinically non-involved lcSSc skin (I). (J–L) Advanced lcSSc skin, double immunofluorescence labelling for CD34 (green) and CD90/Thy-1 (red). Very few telocytes are observed (J and L). Fibroblasts are CD90-positive/CD34-negative (K, L, *arrows*). A CD90-positive vascular wall-resident stem cell niche is scarcely surrounded by telocytes (L, *arrowheads*). Scale bars are indicated in each panel.

**Fig. 4:**
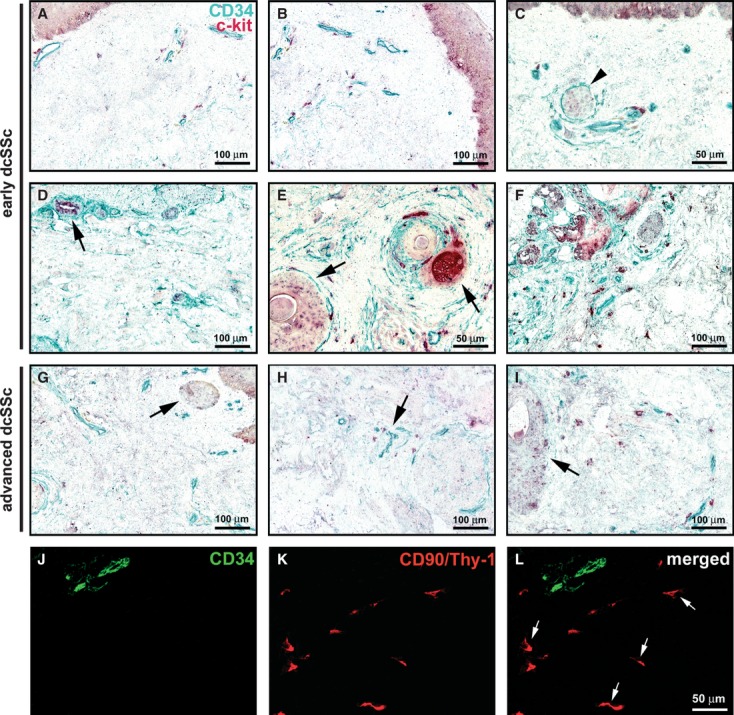
Diffuse cutaneous systemic sclerosis (dcSSc) skin, immunohistochemistry. (**A**–**I**) Double immunoenzymatic labelling for CD34 and c-kit with horseradish peroxidase (green) and alkaline phosphatase (red) detection systems respectively. Everywhere in the dermis, telocytes and endothelial cells are CD34-positive, mast cells are c-kit-positive. Melanocytes (A–C) and epithelial cells of sebaceous (E and G) and sweat (D and F) glands display c-kit-immunoreactivity. (A–F) Early dcSSc skin. Telocytes are very few or absent in the papillary and reticular dermis (A and B). (C) Some telocytes are observed around a nerve (*arrowhead*) and microvessels. Telocytes form a single discontinuous layer surrounding hair follicles and sebaceous glands (E, *arrows*), and eccrine sweat glands (D, *arrow* and F). (G–I) Advanced dcSSc skin. Telocytes are almost completely absent in the papillary and reticular dermis (G–I), in the connective tissue surrounding skin adnexa (G and I, *arrows*) and around occluded microvessels (H, *arrow*). (**J**–**L**) Advanced dcSSc skin, double immunofluorescence labelling for CD34 (green) and CD90/Thy-1 (red). Two microvessels display CD34-positive endothelial cells (J and L). Telocytes are not identifiable, while several fibroblasts are present (K and L, *arrows*). Scale bars are indicated in each panel.

Telocytes and endothelial cells were CD34-positive, and mast cells were c-kit-positive also in skin biopsies from patients with SSc (Figs 3 and 4). However, a striking reduction in telocytes was found in clinically affected skin of SSc patients, with relevant differences according to disease subsets and stages (Figs 3 and 4).

*lcSSc*. In early lcSSc skin, telocytes were almost completely absent from the papillary dermis and reduced in some areas of the reticular dermis, with a patchy distribution (Fig. 3A–D). On the contrary, these cells were preserved around arterioles and nerves, hair follicles, sebaceous glands and eccrine sweat glands (Fig. 3D–F). Moreover, most of the telocytes scattered between collagen bundles of the reticular dermis appeared enlarged in shape (Fig. 3C).

In advanced lcSSc, the loss of telocytes extended to the reticular dermis and the connective tissue around most of the adnexal structures and occluded microvessels (Fig. 3G). Conversely, these cells were still preserved around the secretory and excretory portions of sweat glands in the deep reticular dermis (Fig. 3H). Of note, a normal distribution of telocytes was present in clinically non-involved skin biopsies from lcSSc patients (Fig. 3I).

Double labelling for CD34 and CD90 allowed to confirm the severe reduction and loss of telocytes, and also to evidentiate the presence of numerous CD90-positive fibroblasts in the affected dermis of lcSSc patients (Fig. 3J–L). In addition, telocytes were rarely seen to surround the CD90-positive vascular wall-resident stem cell niches, especially in advanced lcSSc (Fig. 3L).

*dcSSc*. In clinically affected skin from early dcSSc patients, telocytes were very few or absent in both the papillary and reticular dermis (Fig. 4A and B), while some telocytes were still observed around nerves and microvessels (Fig. 4C and D). Moreover, these cells often formed a single discontinuous layer surrounding hair follicles, sebaceous glands and eccrine sweat glands (Fig. 4D–F).

The loss of telocytes was even more severe in advanced dcSSc skin, where they were almost completely absent also in the connective tissue surrounding skin adnexa and around microvessels displaying an occluded lumen (Fig. 4G–I).

Double staining for CD34 and CD90 confirmed either the absence of telocytes or the presence of numerous fibroblasts in dcSSc skin (Fig. 4J–L). CD90-positive vascular wall-resident stem cell niches could not be detected in skin biopsies from the majority of dcSSc patients, especially in the advanced stage.

**Fig. 5:**
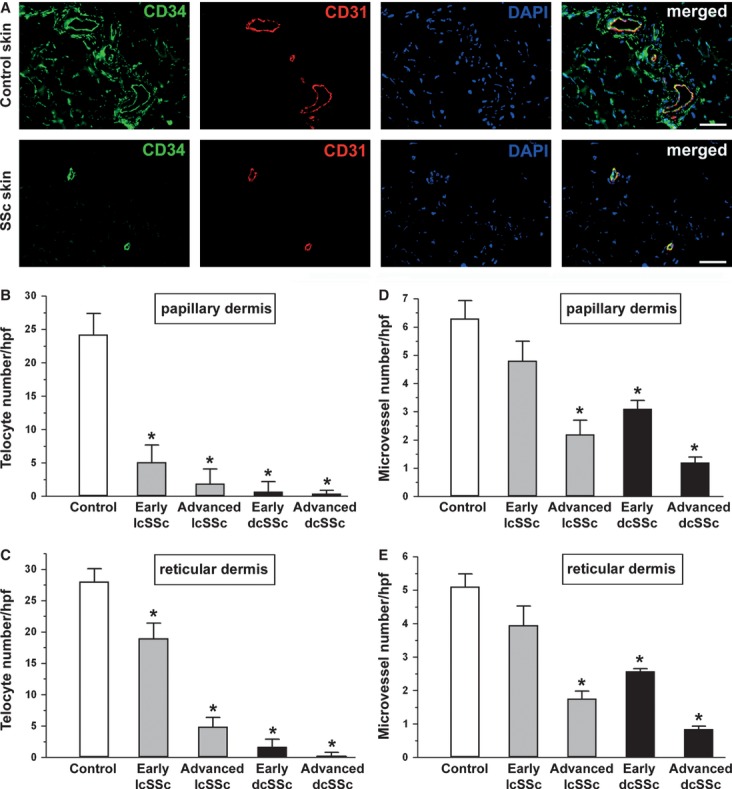
Quantitative analysis of telocytes and microvessels in skin sections from controls and systemic sclerosis (SSc) patients double-immunolabelled for CD34 (green) and CD31 (red) and counterstained with DAPI (blue) for nuclei. (**A**) Representative photomicrographs from control and clinically affected SSc skin samples are shown. Scale bar = 50 μm. CD34-positive/CD31-negative spindle-shaped cells (telocytes) and CD34/CD31-double-positive microvessels were counted in 10 randomly chosen microscopic high-power fields (hpf; 40× original magnification) of the papillary dermis (**B** and **D**) and 10 hpf of the reticular dermis (**C** and **E**) per sample. Only the cells with well defined nuclei were counted. Data are represented as mean ± SD. *P < 0.05 *vs* control (by Student's *t-*test). Limited cutaneous SSc: lcSSc; diffuse cutaneous SSc: dcSSc.

As shown in Figure 5, quantitative analysis demonstrated a significant reduction in the number of telocytes in the papillary and reticular dermis of lcSSc and dcSSc patients, both in early and advanced disease stages, compared with healthy controls. Interestingly, in SSc dermis the reduction in the number of telocytes was paralleled by the progressive reduction in the number of microvessels (Fig. 5). Conversely, no significant difference in the number of fibroblasts between SSc patients and controls could be detected (data not shown).

### Transmission electron microscopy

#### Normal skin

**Fig. 6:**
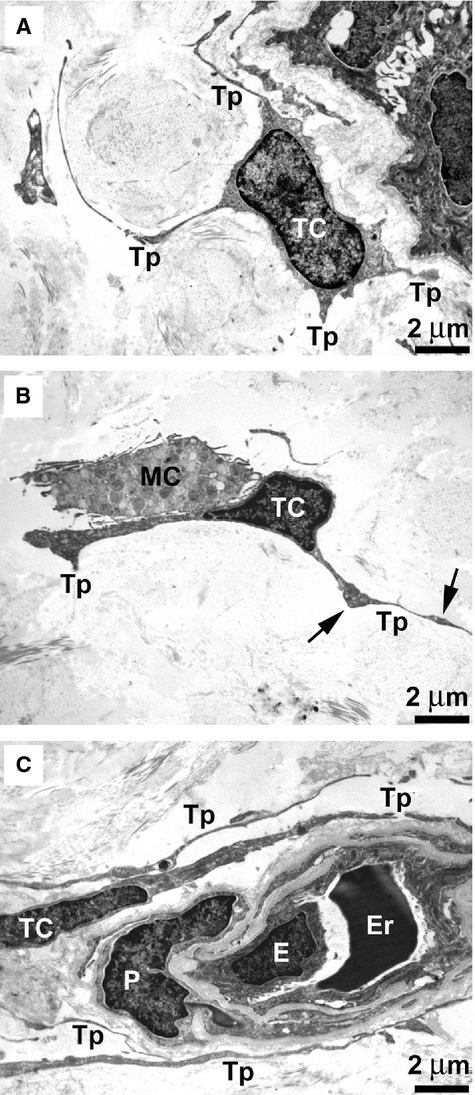
Normal skin, transmission electron microscopy. (**A**–**C**) In both the papillary dermis (A) and reticular dermis (B and C), telocytes have a small cell body and very long and thin processes (telopodes) that are collagen-embedded or lining elastic fibres. Telocytes lack a basal lamina and have a scarce cytoplasm surrounding the nucleus with few mitochondria and cisternae of endoplasmic reticulum, and a small Golgi apparatus. Telopodes display a moniliform aspect due to the alternation of thin segments (podomers) and dilated segments (podoms) oval or triangular in shape (B, *arrows*). (B) A telocyte is in contact with a mast cell. (C) Telocytes are closely associated with each other. The telopodes of perivascular telocytes encircle the basal lamina of a blood microvessel, pericytes are embedded in the vessel basal lamina (C). TC: telocyte; Tp: telopode; MC: mast cell; E: endothelial cell; Er: erythrocyte; P: pericyte. Scale bars are indicated in each panel.

Consistent with previous studies 32, 33, interstitial cells with ultrastructural features of telocytes were identified in the papillary and reticular dermis of normal skin biopsies (Fig. 6). Telocytes appeared as elongated cells with a slender body and two or three long and thin cellular processes, named telopodes, that were usually collagen-embedded or lining elastic fibres (Fig. 6A–C). These cells lacked a basal lamina and had a scarce cytoplasm surrounding the nucleus with few mitochondria and cisternae of rough and smooth endoplasmic reticulum, and a small Golgi apparatus (Fig. 6A–C). Telopodes had an uneven calibre and exhibited a moniliform aspect due to the alternation of thin segments, named podomers, and dilated segments oval or triangular in shape, named podoms, which accommodated mitochondria, endoplasmic reticulum and caveolae (Fig. 6A–C). The cell body and processes of telocytes were often closely associated with or even in contact with each other and with other stromal cells, such as fibroblasts and mast cells (Fig. 6B). Moreover, telocytes and telopodes were often observed around blood vessels and nerve bundles (Fig. 6C). The perivascular telocytes were separated from the endothelial cells by pericytes, and their telopodes formed an almost continuous layer that intimately encircled the vessel basal lamina (Fig. 6C).

#### SSc skin

**Fig. 7:**
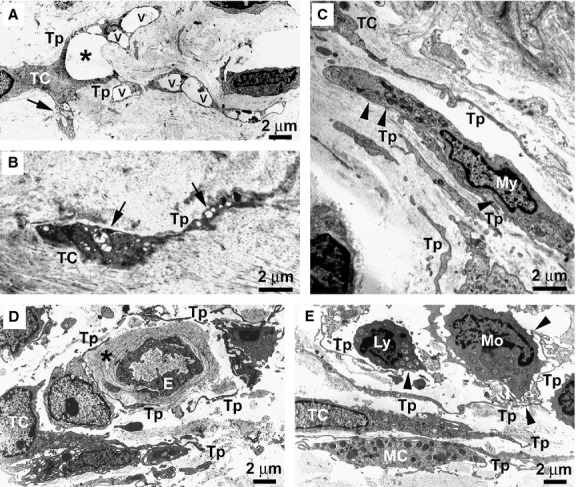
Limited cutaneous systemic sclerosis (lcSSc) skin, transmission electron microscopy. (**A**) Early lcSSc. A telocyte displaying an enlarged shape, due to the presence of large vacuoles (v) in its telopodes, surrounds an area of dermal oedema (*asterisk*). Both normal mitochondria and swollen mitochondria with a clear matrix and few cristae (*arrow*) are identifiable in the cytoplasm. (**B**) Advanced lcSSc. A degenerating telocyte entrapped in the fibrotic extracellular matrix shows numerous swollen mitochondria (*arrows*). The cytoplasm is dark and contains vacuoles and lipofuscinic bodies. (**C**) Early lcSSc. Telocytes and telopodes displaying a normal morphology are present in the close vicinity of or even in contact with a myofibroblast which shows a large body rich in rough endoplasmic reticulum, mitochondria and myofilaments. Subplasmalemmal focal densities are evident (*arrowheads*). (**D**) Early lcSSc. Some telocytes and telopodes with a normal morphology are present around a blood vessel displaying a patent lumen. The vessel basal lamina is markedly thickened (*asterisk*). (**E**) Early lcSSc. Normal telocytes with very long and convoluted telopodes surround a perivascular inflammatory infiltrate composed of monocytes and lymphocytes. Telopodes establish cell-to-cell contacts with inflammatory cells (*arrowheads*). A mast cell is also in contact with telopodes. TC: telocyte; Tp: telopode; My: myofibroblast; E: endothelial cell; Ly: lymphocyte; Mo: monocyte; MC: mast cell. Scale bars are indicated in each panel.

**Fig. 8:**
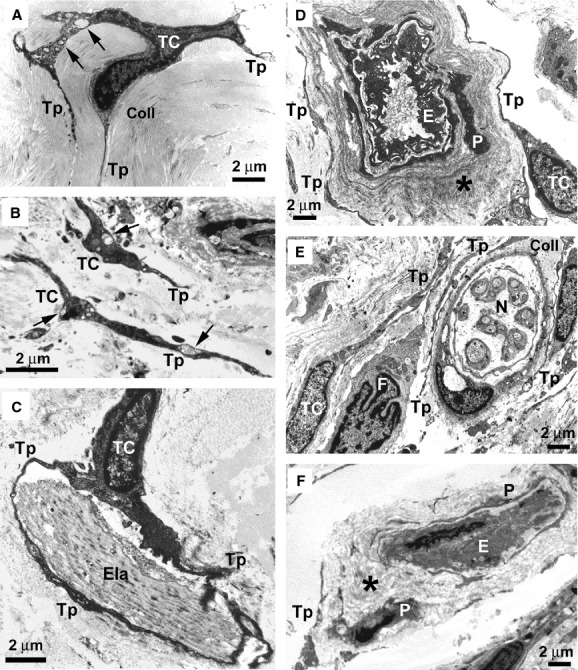
Diffuse cutaneous systemic sclerosis (dcSSc) skin, transmission electron microscopy. (**A**) Early dcSSc. A telocyte with a small perinuclear cytoplasm and slender telopodes is embedded in a matrix composed of closely packed collagen bundles. Swollen mitochondria and vacuoles (*arrows*) are present in the cytoplasm. (**B**) Advanced dcSSc. Telocytes and telopodes embedded in the fibrotic extracellular matrix show features of degenerating cells. The cytoplasm is dark and contains swollen mitochondria (*arrows*), vacuoles and lipofuscinic bodies. Many cell debris are evident. (**C**) Early dcSSc. A telocyte displaying a normal morphology embraces with telopodes a large and abnormal elastin fibre. (**D**) Early dcSSc. Normal telocytes surround the thickened basal lamina (*asterisk*) of a blood vessel displaying a patent lumen. (**E**) Early dcSSc. Telocytes with a normal morphology are evident around nerve bundles. Abundant collagen fibres separate telopodes from the nerve bundle. A fibroblast is in the close vicinity of a telocyte and is surrounded by telopodes. (**F**) Advanced dcSSc. Telocytes are not identifiable around an occluded microvessel. Only a few cell debris are observed. The vessel basal lamina is markedly thickened (*asterisk*). TC: telocyte; Tp: telopode; Coll: collagen; Ela: elastin; E: endothelial cell; P: pericyte; N: nerve; F: fibroblast. Scale bars are indicated in each panel.

Telocytes were severely reduced in the dermis of SSc patients, and most of them exhibited ultrastructural abnormalities (Figs 7 and 8). However, a few normal telocytes were also present in the majority of SSc patients (Figs 7 and 8). Fibroblasts, myofibroblasts, mast cells and macrophages were present, but only a few fibroblasts showed ultrastructural changes, in particular in advanced dcSSc patients.

*lcSSc*. Most of the telocytes embedded in the extracellular matrix displayed ultrastructural abnormalities which, however, were different between the two stages of lcSSc. In early lcSSc, telocytes usually surrounded with their processes oedematous areas of the dermis (Fig. 7A). Moreover, their cell body and telopodes displayed an enlarged shape due to the presence of large vacuoles, and some mitochondria were swollen and had a clear matrix and few cristae (Fig. 7A). In advanced lcSSc skin, many telocytes were entrapped between tightly packed and irregularly distributed collagen bundles, and had ultrastructural features of degenerating cells, such as loss of organelles, numerous swollen mitochondria, lipofuscinic bodies and cytoplasmic vacuolization (Fig. 7B). On the contrary, in both stages of lcSSc, normal telocytes were sometimes observed in the close vicinity of or even in contact with myofibroblasts, which could be easily distinguished from the telocytes because they had a larger body particularly rich in rough endoplasmic reticulum, mitochondria and myofilaments, lacked telopodes, and formed fibronexus junctions with the extracellular matrix (Fig. 7C). Moreover, some telocytes with a normal morphology were also observed around nerve bundles, and around the blood vessels that still displayed a patent lumen despite a markedly thickened basal lamina (Fig. 7D). Conversely, telocytes were absent or degenerated around the occluded microvessels, especially in the advanced stage. Finally, in early lcSSc skin very long and convoluted telopodes surrounded perivascular inflammatory infiltrates, mainly composed of monocytes and lymphocytes (Fig. 7E). These telopodes often established cell-to-cell contacts with inflammatory cells (Fig. 7E).

**Fig. 9:**
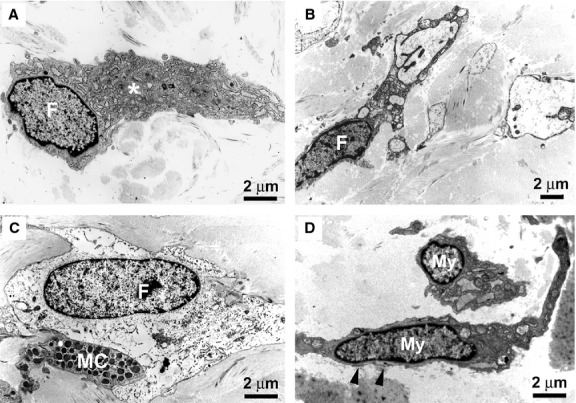
Diffuse cutaneous systemic sclerosis (dcSSc) skin, transmission electron microscopy. (**A**) Early dcSSc. An activated fibroblast displays a particularly extended cytoplasm containing an abundant rough endoplasmic reticulum and a large Golgi apparatus (*asterisk*). (**B** and **C**) Advanced dcSSc. Some fibroblasts embedded in the fibrotic extracellular matrix show ultrastructural features suggestive of a functional exhaustion, such as extremely dilated endoplasmic reticulum cisternae (B) or a clear and empty cytoplasm (C). Note a mast cell in close contact with the fibroblast (C). (**D**) Advanced dcSSc. Myofibroblasts show a large body with short and thick processes and are rich in cisternae of rough endoplasmic reticulum, mitochondria and myofilaments. Subplasmalemmal focal densities are evident (*arrowheads*). F: fibroblast; MC: mast cell; My: myofibroblast. Scale bars are indicated in each panel.

*dcSSc*. As expected considering the marked reduction in CD34-positive spindle-shaped cells, under electron microscopy very few telocytes were found in the skin of dcSSc patients. However, similar to the findings in lcSSc, both normal and altered telocytes could be identified (Fig. 8). In early dcSSc skin, telocytes with a small perinuclear cytoplasm and slender telopodes displaying ultrastructural abnormalities, such as swollen mitochondria and vacuoles, were observed far to each other scattered among closely packed collagen bundles (Fig. 8A). In advanced dcSSc skin, the rarely seen telocytes were embedded in the fibrotic extracellular matrix and showed most severe ultrastructural abnormalities (loss of organelles, numerous swollen mitochondria, lipofuscinic bodies, cytoplasmic vacuolization; Fig. 8B). Often only cell debris could be detected (Fig. 8B). In early dcSSc, some telocytes displaying a normal morphology were observed to embrace with their processes large and abnormal aggregates of elastin fibres (Fig. 8C). Moreover, normal telocytes surrounded the thickened basal lamina of blood vessels still displaying a patent lumen (Fig. 8D) and nerve bundles (Fig. 8E). On the contrary, no or degenerating telocytes were seen around occluded microvessels in both stages, although more frequently in advanced dcSSc (Fig. 8F). In all dcSSc skin biopsies, fibroblasts exhibited a particularly extended cytoplasm containing an abundant rough endoplasmic reticulum and a large Golgi apparatus (Fig. 9A). However, in the advanced disease stage, some fibroblasts had ultrastructural features suggestive of a functional exhaustion (Fig. 9B and C). Myofibroblasts were present in both stages and showed no morphological alterations (Fig. 9D).

## Discussion

In this study, by an integrated immunohistochemical and ultrastructural approach, we clearly demonstrate that telocytes are a distinct stromal cell population of human dermis, and that they are severely damaged progressively disappearing from the clinically affected skin of patients with SSc.

In the last years, numerous reports have shown the existence of a novel and peculiar stromal (interstitial) cell type, the telocyte, in various mammalian and human cavitary and non-cavitary organs 14, 16–33. Herein, we confirm that telocytes are present in human dermis, where they are mainly recognizable for their typical ultrastructural features 33, and are immunophenotypically characterized by the expression of CD34, a marker shared with endothelial cells. However, to better characterize the immunophenotype of the telocytes and exclude their possible overlap with other dermal cell types, we carried out a series of double immunoreactions using anti-CD34 antibodies in combination with antibodies against several cell-specific antigens. The present findings indicate unequivocally that, in human skin, telocytes are a distinct stromal cell population immunophenotypically negative for CD31/PECAM-1 (endothelial cells), α-SMA (myofibroblasts, pericytes and vascular smooth muscle cells), CD11c (dendritic cells and macrophages) and CD90/Thy-1 (fibroblasts). In addition, unlike mast cells, dermal telocytes do not express c-kit/CD117. Our immunohistochemical data are in line with the view that, although telocytes might show different immunohistochemical profiles among different organs and even in the same organ examined, at present, CD34 labelling remains the best available choice for telocyte identification 16.

For the first time, we show that telocytes display ultrastructural damages, are significantly reduced and progressively disappear from SSc skin. In agreement with previous studies 32, 33, we have observed that telocytes in normal skin are organized to form three-dimensional networks distributed among collagen bundles and elastic fibres throughout the whole dermis. Moreover, some telocytes appeared concentrated around vascular structures, nerves and skin adnexa (hair follicles, sebaceous and eccrine sweat glands). In agreement with a previous report on a selective disappearance of ‘CD34-positive spindle-shaped cells’ from the skin lesions of SSc 40, we observed a striking reduction in telocytes in the affected skin of all our SSc patients. Our data show that the telocyte loss evolves differently according to the SSc subsets and stages. In early lcSSc, telocytes are absent from the papillary dermis and patchily reduced in some areas of the reticular dermis. In advanced lcSSc, the loss of telocytes is severe also in the reticular dermis and the connective tissue surrounding the adnexal structures. In the early stage of dcSSc, which is characterized by a more rapid disease progression 1, telocytes are very few or absent in both the papillary and reticular dermis and around most of the skin adnexa. In advanced dcSSc, telocytes are almost completely disappeared. Thus, as previously shown for the modifications of skin innervation and microcirculation 10, the progression in telocyte reduction occurs earlier and is more severe in dcSSc than in lcSSc. Moreover, this progressive reduction in telocytes occurs in parallel with the severity of their ultrastructural abnormalities. Indeed, telocyte ultrastructural alterations (swollen mitochondria, cytoplasmic vacuolization and presence of lipofuscinic bodies) may indicate a cellular degenerative process already detectable in early dcSSc and more marked in the advanced stage of both SSc subsets.

Different mechanisms might be responsible for the damage and loss of telocytes in clinically affected skin from SSc patients. The chronic ischaemic microenvironment of SSc skin, characterized by low levels of oxygen, generation of reactive oxygen species and scarcity of nutrients 6, 7, 41, may compromise the telocyte metabolism and cause profound cell sufferance. Indeed, in SSc dermis the reduction in the number of telocytes was paralleled by the reduction in microvessel density. Of note, the loss of telocytes was dramatic already in the early disease stages, while that of microvessels proceeded more slowly (see Fig. 5). Furthermore, the hypothesis of an ischaemic injury is strongly supported by the presence in telocytes of numerous swollen mitochondria and extensive cytoplasmic vacuolization. The most severely affected telocytes were those embedded in the fibrotic extracellular matrix and those surrounding occluded microvessels, while the telocytes found around patent microvessels displayed a normal morphology, even in the advanced disease stages. With disease progression, the more severe and extended damage of telocytes might be caused by their entrapment in a poorly permeable matrix due to the overproduction and accumulation of abnormal collagen and elastic fibres. It remains, however, to be elucidated why telocytes appear to be more sensitive to ischaemia than other stromal cell types, such as fibroblasts, myofibroblasts and mast cells. In fact, myofibroblasts and mast cells showed normal features in SSc skin, and a few fibroblasts had ultrastructural abnormalities in dcSSc only, especially in the advanced stage. Finally, when considering the autoimmune background of SSc 1, 2, we cannot exclude the possibility that telocytes might be important/specific target cells of autoantibodies. However, such hypothesis seems little supported as telocytes were unaffected in clinically non-involved skin biopsies from SSc patients.

The progressive ultrastructural damage and loss of telocytes might have important pathophysiologic implications in SSc. According to the functions that have been proposed for these cells 16, different scenarios may be suggested. First, the loss of telocytes could contribute to the altered three-dimensional organization of the extracellular matrix found in SSc skin. As suggested in the developing and adult heart 42, 43, by their long cytoplasmic processes telocytes might act as supporting cells and form a scaffold to guide the migration of other cells, including precursor cells, to define the correct three-dimensional organization of an organ during pre-natal life, or its repair or renewal in post-natal life 16. Similarly, the three-dimensional network of telocytes might guide the correct assembly of the extracellular matrix compartment within connective tissues. Indeed, we and others 32, 33 have observed that in normal dermis telopodes are usually collagen-embedded or lining elastic fibres. Interestingly, in SSc skin some telocytes were found to embrace with their processes very large and abnormal aggregates of elastin and collagen fibres, likely in the attempt to limit their spreading into the interstitium. Similar findings were observed in human heart amyloidosis, where amyloid deposits were located in interstitial recesses surrounded by long and slender telocyte processes 44. The second hypothesis could be that the loss of telocytes might favour the abnormal activation of fibroblasts and mast cells in SSc skin 5, 45. Indeed, it has been suggested that telocytes are involved in intercellular signalling that could influence the transcriptional activity of neighbouring cells, either directly, by cell-to-cell contacts, or indirectly, by shedding microvesicles and exosomes or secreting paracrine signalling molecules, including microRNAs 16, 33, 46, 47. Thus, telocytes may convert the interstitium into an integrated system that contributes to maintaining organ homeostasis 33. As previously reported by others 32, 33, we presently observed in the normal dermis intercellular contacts between telocytes and fibroblasts, myofibroblasts and mast cells. Therefore, it is tempting to speculate that telocytes could be involved in the maintenance of the normal skin homeostasis by controlling fibroblast, myofibroblast and mast cell activity. In SSc patients, this control is likely altered due to the telocyte reduction and loss. Finally, in SSc skin, the disappearance of telocytes could impair stem cell-mediated tissue regeneration. Indeed, increasing evidence indicates that telocytes may cooperate with stem cells to form tandem cell structures 15, which are found in stem cell niches of various organs and have been implicated in tissue regeneration and/or repair 18, 25, 31, 33, 42, 47. Functional stem cell niches have been described throughout all layers of human skin, including the hair follicle bulge, interfollicular epidermis, dermal papillae and the so-called perivascular or vascular wall-resident stem cell niches 36–39. Recently, it has been observed that telocytes surround stem cell niches in human skin and proposed that these cells might play the role of ‘nurse’ cells for adjacent stem cells 33. In agreement with these data, we also observed telocytes surrounding stem cell niches in the normal skin, but, interestingly, they were rarely seen in SSc skin. Moreover, we could not detect vascular wall-resident stem cell niches in most of the dcSSc skin biopsies. Telocyte loss, therefore, might contribute to the depletion of functional stem cell niches and impair skin regeneration and/or repair in SSc patients. Interestingly, in a recent study telocytes were found to be decreased during experimental myocardial infarction, and transplantation of cardiac telocytes in the infarcted and border zones of the heart decreased the infarction size and improved myocardial function 48. Further studies will be required to investigate the functional relationship between dermal telocytes and stem cell niches and the potential therapeutic utility of telocyte transplantation for the treatment of SSc.

In conclusion, the present findings demonstrate that in human skin telocytes are a distinct stromal cell population (CD34-positive and CD31/α-SMA/CD11c/CD90/c-kit-negative), and that dermal telocytes are selectively damaged in SSc, a prototypic fibrotic disorder. In SSc skin, the progressive reduction and loss of telocytes might (i) contribute to the altered three-dimensional organization of the extracellular matrix, (ii) reduce the control of fibroblast, myofibroblast and mast cell activity, and (iii) impair skin regeneration and/or repair. However, the pathogenetic mechanisms underlying the loss of dermal telocytes and their functional consequences in SSc need to be further investigated. It will be also of high interest to ascertain whether telocyte damage and disappearance is restricted to the skin or it may also occur in the internal organs targeted by the SSc fibrotic process, such as the lung, heart and gastrointestinal tract 2–4, 49–51. Finally, it is important to investigate the tissue distribution and the ultrastructural features of telocytes in other fibrotic disorders.
